# Residents as learning facilitators inside and outside of interprofessional education: a faculty development program in postgraduate pediatric training

**DOI:** 10.3389/fmed.2025.1491177

**Published:** 2025-02-06

**Authors:** Philipp A. Müller, Christine Straub, Andrea Heinzmann, Thorsten Langer, Sebastian F. N. Bode, Jan Griewatz, Christian Kimmig, Sebastian Friedrich

**Affiliations:** ^1^Department of General Pediatrics, Adolescent Medicine and Neonatology, Medical Centre, Faculty of Medicine, University of Freiburg, Freiburg, Germany; ^2^Department of Neuropediatrics and Muscle Disorders, Medical Centre, Faculty of Medicine, University of Freiburg, Freiburg, Germany; ^3^Department of Pediatrics and Adolescent Medicine, Ulm University Medical Center, Ulm University, Ulm, Germany; ^4^Tübingen Institute for Medical Education, Faculty of Medicine, University of Tübingen, Tübingen, Germany

**Keywords:** Interprofessional education, resident-as-teachers, train the trainer, faculty development, core competencies for medical teachers

## Abstract

**Introduction:**

Changing learning environments in health professions are an important challenge of interprofessional education (IPE). When students experience IPE activities during their undergraduate training, they are often guided by trained learning facilitators. Students still spend more time in non-IPE settings, often guided by residents. Residents rarely undergo specific training for core teaching competencies that are crucial in both IPE and non-IPE contexts. At our pediatric hospital, some residents were trained as learning facilitators on an interprofessional training ward. To bridge the gap between IPE and non-IPE learning facilitation for the other residents, we developed the resident-as-teachers course “How to teach pediatrics.”

**Methods:**

“How to teach pediatrics” was implemented as a 4-week blended learning program based on the framework of Core Competencies for Medical Teachers (KLM). The intended learning outcomes were to reflect on residents’ role modelling and professionalism as well as personal teaching practice, emphasize learner centeredness and foster social and communicative competencies. Participants self-assessed their teaching competencies pre/post-course using a validated questionnaire (FKM_L). Oral feedback was gathered by group reflection and qualitative feedback by open-ended survey questions.

**Results:**

26 residents participated in the course, of which *N* = 22 qualified for the pre/post-course self-assessment via the FKM_L (return rate: *n* = 9; 40.9%). Participants reported an increase in the competency fields of “didactical activities in medicine,” “social and communicative competence,” “role model and professional behavior” as well as “reflection and further development of own teaching practice.” Participants evaluated the course overall as “very good,” stated a high learning gain and estimated the course to be a good preparation for teaching students.

**Discussion:**

“How to teach pediatrics” shows the feasibility of integrating faculty development as part of resident training. We observed a self-assessed increase in core competencies for medical teachers after participating in the course. Although more participants need to be included and long-lasting effects still need to be proven, such faculty development programs for learning facilitators might be an opportunity to ensure a more consistent and high-quality learning experience for students in both IPE and non-IPE teaching and learning activities.

## 1 Introduction

Changing learning environments for students in health professions represent one of the most significant challenges in interprofessional education (IPE) (1, 2). Over the past decades, an increasing number of IPE teaching and learning activities have been developed. During these undergraduate training sessions, students are often supported by learning facilitators (3). Given the complex dynamics inherent in IPE courses, there is broad consensus that effective faculty training is essential for IPE. Consequently, learning facilitators must be equipped with teaching competencies that include reflecting on roles and responsibilities, facilitating learning, fostering discussion and team communication and developing a professional identity (4–6). Those competent learning facilitators are crucial as they promote a culture of open communication and active listening, as well as creating a “safe place with space for learning” (7). As a result and necessity, faculty development and the provision of teaching competencies have become areas of growing interest in interprofessional education (3, 8).

While IPE-related training is crucial for IPE-learning facilitators, it may not be sufficient. Structured IPE activities still constitute only a small portion of the overall curriculum for medical students, although students constantly find themselves in non-structured IPE and non-IPE activities during their courses or day-to-day clinical practice. Students still spend more time in non-IPE activities, where they interact with various learning facilitators, many of whom are residents. Those medical experts play a central role in the education of students. Medical students reported that they acquire approximately one-third of their knowledge from residents (9). Thereby residents have always been deeply involved in clinical teaching, dedicating a significant part of their daily work to teaching medical students. Their critical role in teaching both medical students and fellow residents became increasingly clear over the past few decades (10–13). Residents in non-IPE settings are also highly motivated and there is substantial evidence highlighting their importance as learning facilitators (14). However they rarely participate in formal faculty development programs or residents-as-teachers workshops. The majority of residents don‘t receive any standardized teaching training before starting their residency or during residency (15, 16).

While there is a lack of faculty development for residents, several competency frameworks for medical professionals have been established. One model was proposed by the Royal College of Physicians and Surgeons in Canada (CanMEDS) (17). In this framework the role of the “Scholar” emphasizes the responsibility in teaching as a lifelong learner who improves and maintains professional action and behavior through continuous learning. Based on the CanMEDS framework and the “Competencies for Medical Educators” by Srinivasan et al., Görlitz proposed the Core Competencies for Medical Teachers (Kernkompetenzen für Lehrende in der Medizin, KLM) (18, 19). The KLM serves as a guide for the qualification of teaching faculty and supports further advancement of the content, training formats and evaluation of faculty development initiatives and therefore, establishes uniform quality criteria. The KLM outlines a profile of requirements for all teachers in medical education as it defines six competencies for medical teachers, which are equally relevant: educational action in medicine, learner centeredness, social and communicative competencies, role modelling and professionalism, reflection and advancement of personal teaching practice and systems related teaching and learning (18). These competences overlap with the competencies in the IPE context mentioned above, especially regarding learner centeredness, social and communicative competencies, role modelling and professionalism, reflection and advancement of personal teaching practice. The development in those competencies cannot be taken for granted in the increasing complexity of daily healthcare delivery. This underscores the importance of longitudinal faculty development programs, both within IPE and non-IPE, to offer residents the opportunity to further develop their competencies. Residents-as-teachers workshops and faculty development enable residents to acquire essential teaching competencies and core competencies for learning facilitation in both IPE and non-IPE context (20). In our tertiary pediatric hospital, residents have been taken on the role of learning facilitators for medical and nursing students on an interprofessional training ward since 2017 (21–24). In advance they received a train-the-trainer workshop where they participated in sessions providing competencies on communication, feedback, learner centeredness, role modelling and self-reflection (25). These train-the-trainer workshops could only be offered to residents who participated in our interprofessional training ward as learning facilitators. However, the majority of the residents in our tertiary pediatric hospital still have not received any training in teaching competencies or core competencies for learning facilitation. Hence there is still a gap in the training for learning facilitation between IPE and non-IPE in our hospital. To bridge this gap through faculty development, we conceptualized and implemented a residents-as-teachers course named “How to teach pediatrics.” This led to the following research questions:

- How does the “How to teach pediatrics” course influence IPE and non-IPE-related core medical teaching competencies in our tertiary pediatric hospital?
- In which way does our course influence self-perceived core medical teaching competencies in pediatric residents?
- Which aspects of the course influence self-perceived competencies in particular?

## 2 Methods

### 2.1 The residents-as-teachers workshop: “how to teach pediatrics”

To overcome the gap between IPE and non-IPE learning facilitation through faculty development, we conceptualized and implemented a residents-as-teachers course named “How to teach pediatrics.” The course program was developed based on the framework of Core Competencies for Medical Teachers (german: *Kernkompetenzen für Lehrende in der Medizin =* KLM) (18). For the purpose of the workshop “How to teach pediatrics,” we focused on four of the six KLM competencies that overlap with the competency frameworks for learning facilitators on interprofessional training wards. Therefore we focused on learner centeredness, social and communicative competencies, role modelling and professionalism and reflection and advancement of personal teaching practice as we saw the greatest overlaps within these competency fields.

We developed “How to teach pediatrics” according to the principles of constructive alignment (26). Following these principles, intended learning outcomes (ILOs) should be in line with teaching and learning activities and assessment tasks. The main intended learning outcomes for “How to teach pediatrics” were derived from the four KLM competencies mentioned above. Specifically, the intended learning outcomes were:

(1) Residents analyze their role as learning facilitators and their role modeling for undergraduate medical students in an individual or small group setting.
(2) Residents apply principles of student-centered learning, such as considering students’ prior knowledge and fostering a safe learning environment.
(3) Residents implement theory-based approaches in providing structured feedback to undergraduate medical students
(4) Residents reflect their personal teaching practice and advance teaching competency development.

**Figure 1:**
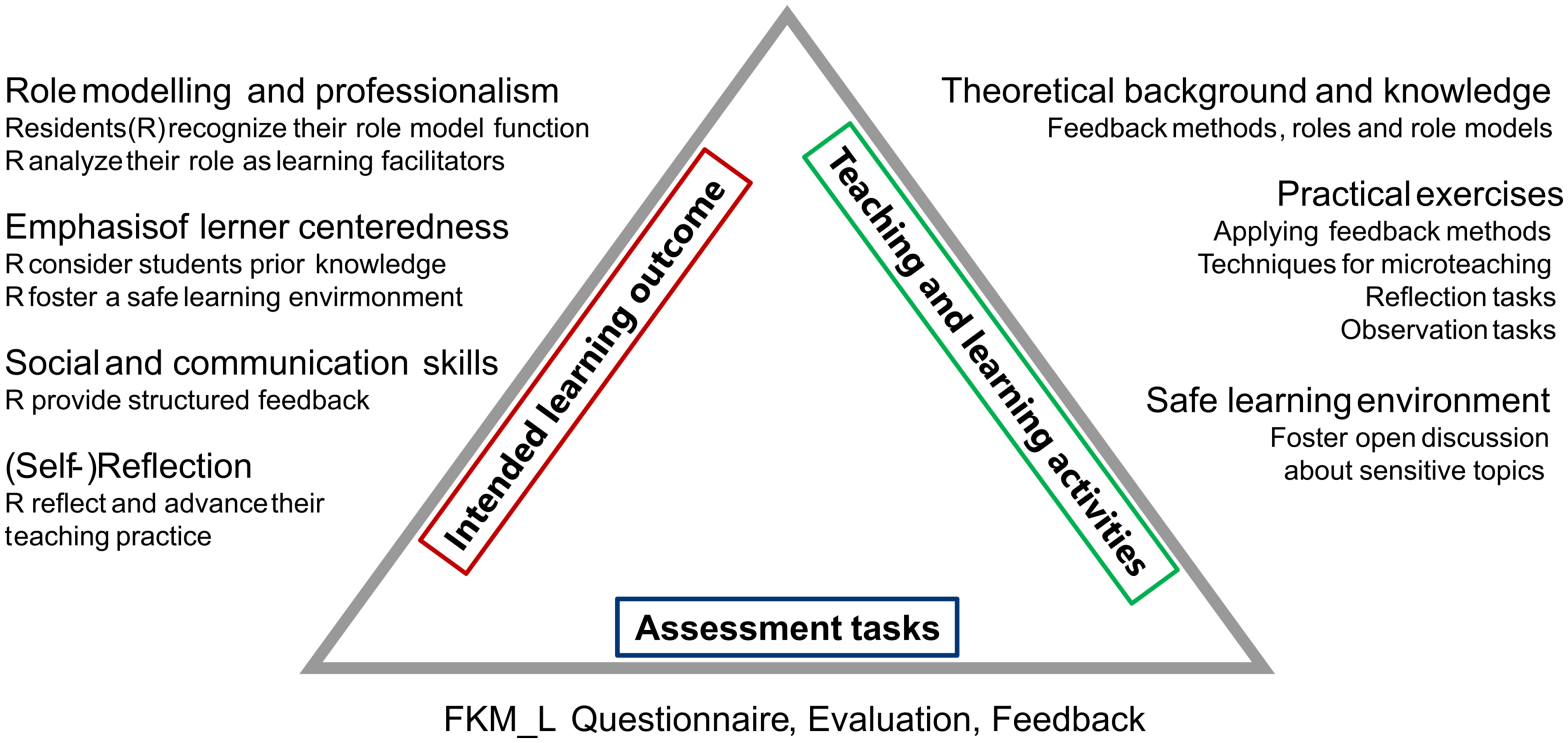
How to teach pediatrics—constructive alignment. Intended Learning Outcome (=ILO, framed in red): Reflection on residents’ role in the domain of role modelling and professionalism, (self-)reflection and development of personal teaching practice, emphasis of learner centeredness and advancement of social and communicative competencies. Teaching and learning activities (framed in green): Mediation of theoretical background and knowledge, practical exercises and creation of a safe learning environment. Assessment (framed in blue): Rating of the participants own teaching competencies pre/post using a validated questionnaire [FKM_L = Freiburg questionnaire for assessing competencies in medicine, teachers (German: Freiburger Fragebogen zur Erfassung von Kompetenzen in der Medizin, Lehrende)], evaluation of the course and direct feedback during the course.

Based on the intended learning outcomes, we developed teaching and learning activities and selected assessment tasks (Figure 1).

“How to teach pediatrics” was developed as a training course that lasts 4 weeks. The course was led by experienced teachers from the medical context. All of them have taken on the role of learning facilitators for medical and nursing students in our interprofessional training ward.

At the start of the course, participants were asked to complete an online preparatory e-learning, which was then used as a flipped classroom activity in the first on-site workshop. In the e-learning, we mainly covered different areas of providing and receiving feedback, including specific techniques. Additionally, participants were given a reflection task about their own past learning experiences from both undergraduate and postgraduate medical training. The reflection task consisted of two parts:

1. “Thinking back to your undergraduate and postgraduate medical training, which teacher stands out for you and why?”
2. “How exactly did this teacher facilitate your learning?”

This reflection task served to relate concepts of role modeling and learning facilitation to participants’ own lived experience. It was designed to facilitate participants’ discussion on the role and importance of being a role model during the first on-site workshop. This workshop also included practical exercises on feedback techniques in role-play activities [e.g., Ask-Tell-Ask method; (27)]. Furthermore, the importance of creating a safe learning environment as a prerequisite for successful social learning was emphasized, based on the participants’ shared reflections. Elements of learner centeredness were discussed, with a particular emphasis on promoting a culture that embraces mistakes. After the course, participants received an observation task:

“In the next 2 weeks, you should be observed by a final year medical student in a situation involving patients and/or parents and receive feedback from them. This situation could involve taking a brief medical history, conducting an examination, or having a conversation.”

**Figure 2:**
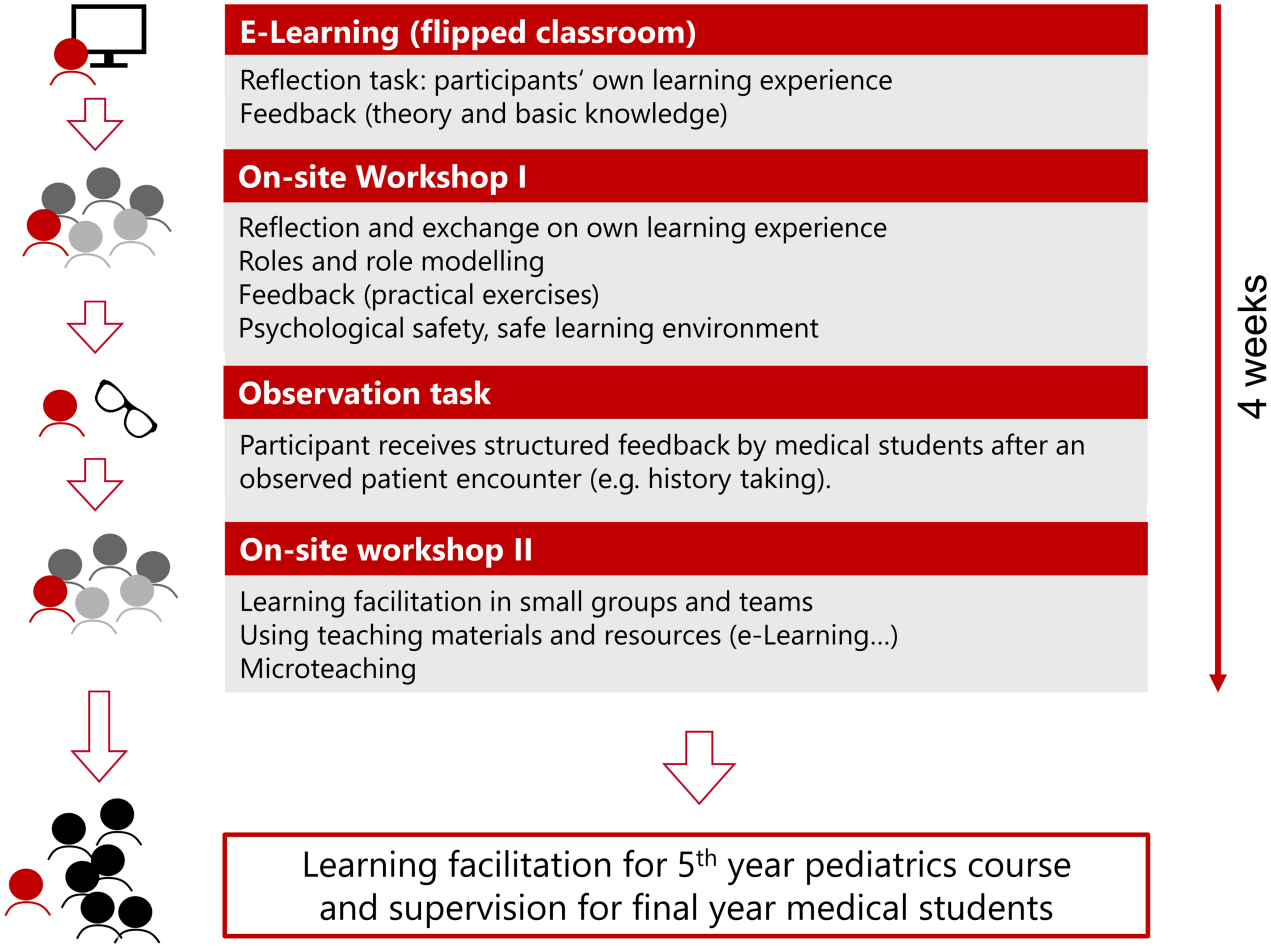
How to teach pediatrics—concept. The red mannequin represents a resident participating in the four-week residents-as-teachers workshop. The dark and light grey mannequins are other participants and the black mannequins are medical students. In the beginning participants go through an e-Learning. The first on-site workshop includes exchange on the reflection exercise, practical exercises and input on creating a safe learning environment. Subsequently participants are observed by medical students and receive structured feedback afterwards. During the second on-site workshop participants share their experiences and discuss aspects of group dynamics, microteaching, group teaching techniques and the teaching materials available at our clinic. After the four-week training, course participants will teach medical students during the 5th year pediatric course and supervise medical students on the wards.

The aim of the observation task was to practice accepting feedback correctly and to model this behavior for students. Medical students in their final year of a six-year course spending rotations on different wards of the hospital were given a structured observation form and then asked to give feedback to residents. Residents approached medical students that were available in their current work environment prior to the second on-site workshop. Medical students were then asked to observe residents in a short day-to-day activity, e.g., taking a history from a patient and parents, or conducting a physical examination. In the workshop, participants shared their experiences with the observation task. This led to a lively discussion around giving and receiving feedback. Additionally, there were sessions on how to deal with group dynamics and small group teaching techniques [e.g., Think-Pair-Share method; (28)]. We also discussed the teaching materials available at our clinic and how to use them to achieve students’ learning goals (Figure 2).

### 2.2 Participants

The course was initially offered to residents of a tertiary pediatric hospital in the first 2 years of training only. Group size was limited to 8 participants, to allow for a close facilitation by the one person teaching the course. For the first two rounds of the course, participants were recruited according to availability during course hours in the afternoon (e.g., not on holiday, available to be absent from the ward for 3 h, no shift work). Participants were informed about their participation via email. 8 and 7 residents took part in the first two rounds of the course. For the third round of the course, some adaptations were made. Firstly, two teaching faculty were available for the course, so the number of participants was raised to a maximum of 15. Secondly, the course was opened to all residents and fellows of the pediatric hospital, as well as pediatricians from outpatient primary care pediatric offices. Primary care pediatricians participated in the course to prepare for a new program, where final year medical students spend 4 weeks of their pediatrics rotation in outpatient primary care pediatric offices. Participation in the course was offered to all residents and fellows via email (around 70 people), with 10 places available. Likewise, primary care pediatricians were invited to the course via email (around 30 people), with 5 places available. Finally, 6 residents, 1 fellow and 4 primary care pediatricians participated in the third round. All participants were asked to give oral feedback at the end of the course and fill in both the course evaluation and the FKM_L questionnaire in both the pre and the post self-assessment.

### 2.3 The FKM-L questionnaire

To measure the achievement of the defined Intended Learning Outcomes (ILOs), we used the FKM_L Questionnaire, which was “developed to capture individual and group-based competency profiles of medical educators” (29). The FKM-L concept is based on the Core Competencies for Medical Teachers [KLM; (18)]. The questionnaire assesses six core competencies of the KLM model through global questions and subareas, enabling medical teachers to understand and reflect on their teaching competencies.

For each competency field of the FKM_L the items of the subareas were summarized with good internal consistency. Across the six core competencies, there are 22 subareas and further subscales, comprising a total of 69 items. All scales were subjected to item analysis. For each item (e.g., “I use different teaching/learning methods in my classes), respondents were asked to rate their approval on a five-item Likert type scale (“Totally agree” to “Do not agree at all”) (29).

**Figure 3:**
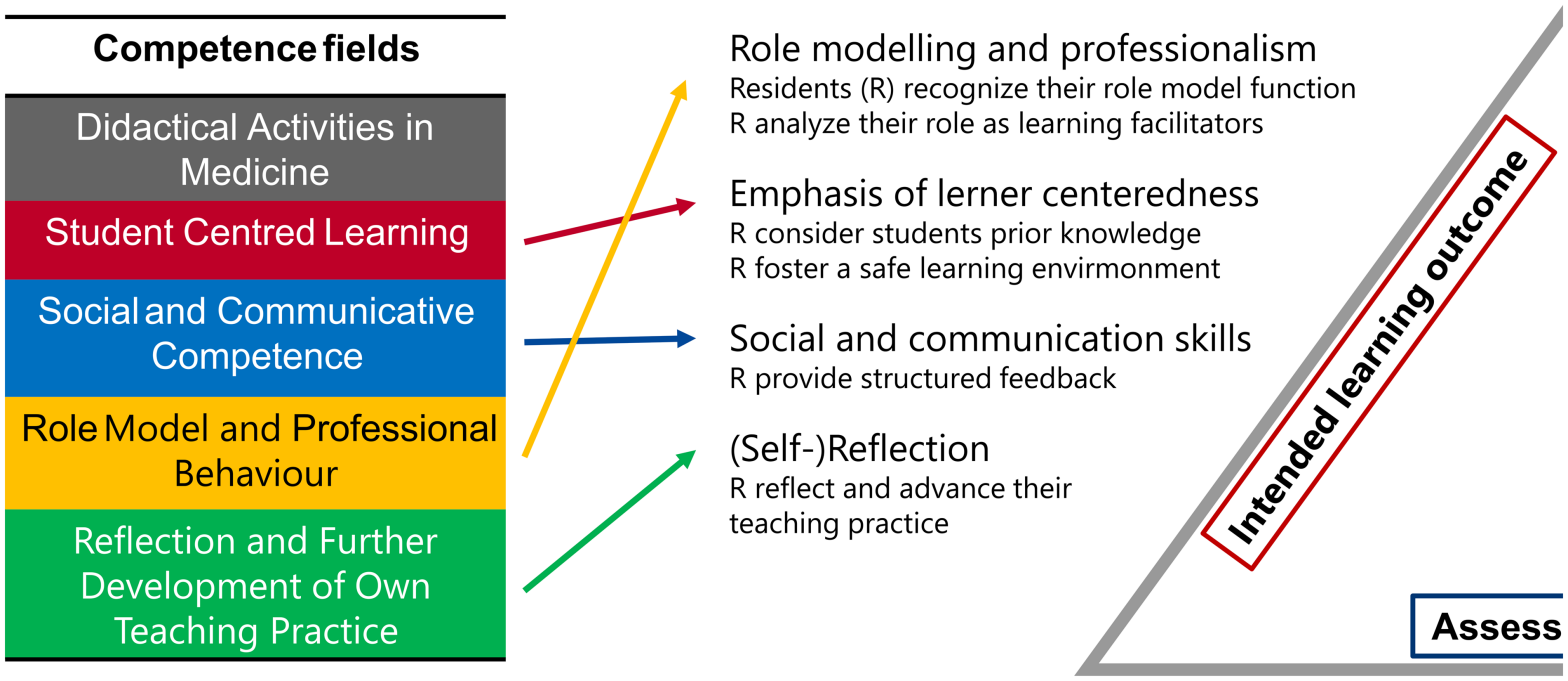
FKM_L Competence fields and intended learning outcome. The five competence fields assessed through the FKM_L questionnaire are presented. The colored arrows indicate the corresponding content of our intended learning outcomes and the core competencies we aim to convey.

For our faculty development program, “How to Teach Pediatrics,” we examined the competency fields of “Learner Centeredness,” “Social and Communication Skills,” “Role Modelling and Professionalism,” and “Reflection and Development of One’s Own Teaching Practice.” Additionally, we were interested in the self-assessment of the competency field “Medical Didactic Skills.” We excluded the subareas “Examination” and “Coherence with Examination Goals” as they were not relevant to our course. We also left out the core competency of “System-Based Learning” since our course aimed to improve individual teaching competencies of the residents (Figure 3).

### 2.4 Course evaluation form

Course evaluation consisted of an online questionnaire with 19 5-point Likert-type items and two open ended questions (“What did you like the most?,” “What could be improved?”). It was based on a modified version of the Maastricht Clinical Teaching Questionnaire, which is used at the local university to evaluate small-group teaching and seminars in medicine (30).

### 2.5 Data collection

Data were collected between 2021 and 2024 during the “How to Teach Pediatrics” courses. During that time, 21 residents, 1 fellow and 4 primary care pediatricians participated in the course. Participants were asked to complete both the course evaluation and the FKM-L questionnaire. The FKM-L was completed both as a pre-assessment and a post-assessment upon the end of the course. Data collection was conducted online via the Unipark platform by Tivian (www.unipark.de, Tivian GmbH, Hürth, Germany) on mobile devices.

### 2.6 Data analysis

Data were analysed in Microsoft Excel. Means and standard deviations for subscales and global items were calculated as described previously (29). Due to small sample size for the FKM_L, only descriptive statistical analysis was conducted. Free-text comments from evaluation forms were extracted and analysed by two authors independently (PAM and SF) and grouped according to positive and negative aspects about the course.

### 2.7 Ethics

All participants gave written informed consent before completing the questionnaire. Completing the FKM_L was not mandatory for participating in “How to teach pediatrics.” The study was approved by the Institutional Review Board of the University of Freiburg (No 21–1300).

## 3 Results

### 3.1 Sample characteristics

26 residents participated in a total of three implementations between November 2021 and April 2024. The course evaluation was completed by 20 of 26 participants (return rate 76.9%). For the pre/post self-assessment via FKM_L questionnaire, analysis was limited to hospital-based doctors (i.e., residents and fellows), lowering the number of possible respondents to *N* = 22. The response rate was n = 9, return rate 40.9%. 44.4% of these nine participants were female, 77.8% were between 25 and 30 years old and the mean of years working in hospital was 2 years. Full sample characteristics can be found in Supplementary data.

### 3.2 Self-assessment: core competencies for medical teachers

**Figure 4:**
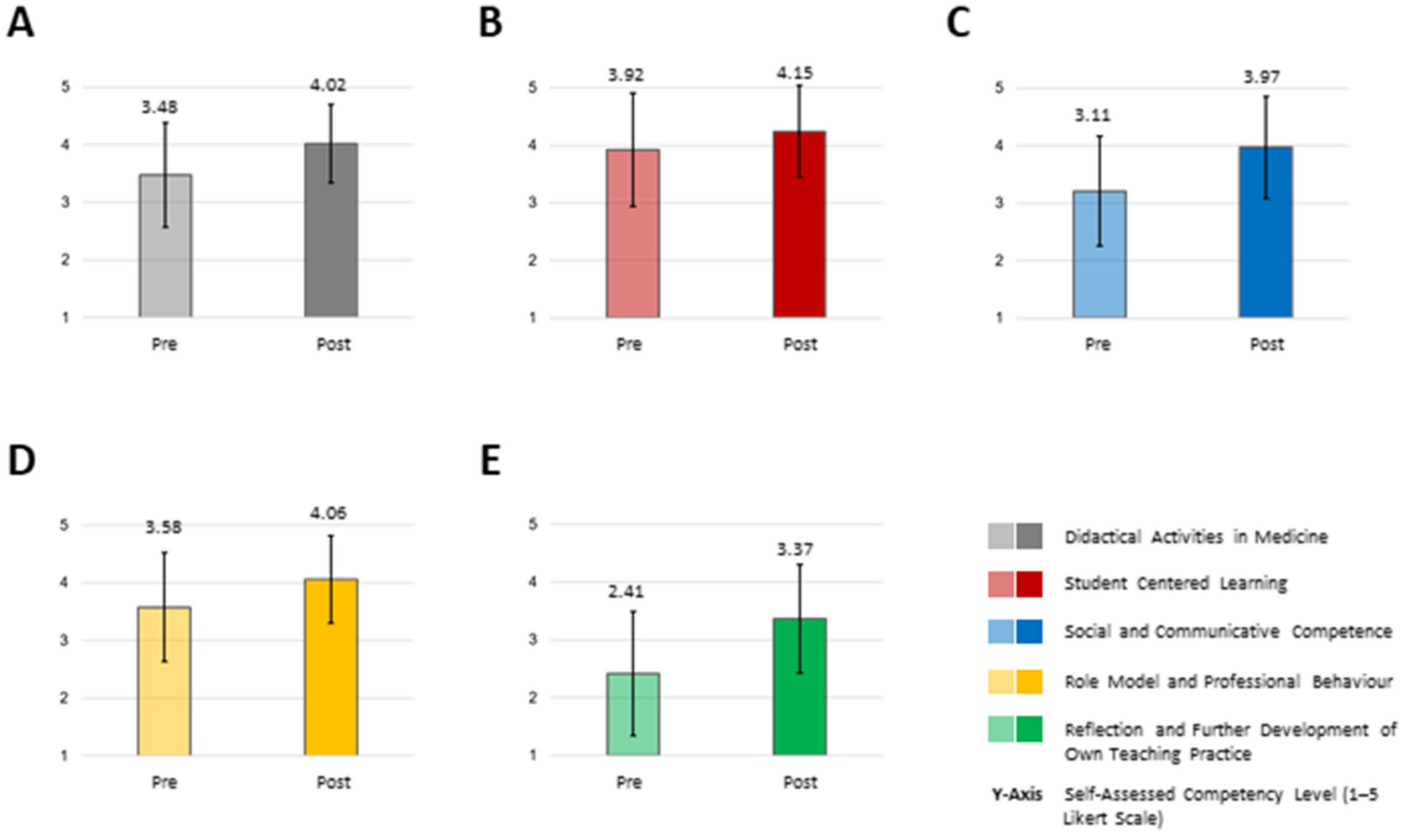
Competence fields pre/post “How to teach pediatrics.” The results show the self-perceived teaching competencies pre/post our teaching course using the FKM_L questionnaire (**A**: “Didactical Activities in Medicine”; **B**: Student Centered Learning”; **C**: “Social and Communicative Competence”; **D**: “Role model and Professional Behavior” and **E**: “Reflection and Further Development of Own Teaching Practice”). The colored bars (pre = transparent colored, post = strong colored) show the mean values based on a five-point Likert scale with 1 indicating low level of approval, meaning low competency level and 5 indicating a high level of approval, therefore high level of competency. Narrow lines represent the standard deviation. *n* = 9.

Due to small sample size for the FKM_L, we describe trends and conduct descriptive statistical analysis in the following. We saw an increase in all five core competencies which we recorded with the FKM_L questionnaire. Participants reported an increase in their self-perceived competencies in “didactical activities in medicine” (mean: pre: 3.48; SD: 0.91 versus post: 4.02; SD: 0.68), “student centered learning” (mean: pre: 3.92; SD: 0.99 versus post: 4.15; SD: 0.80), “social and communicative competence” (mean: pre: 3.11; SD: 0.95 versus post: 3.97; SD: 0.89), “role model and professional behavior” (mean: pre: 3.58; SD: 0.94 versus post: 4.06; SD: 0.76) and “reflection and further development of own teaching practice (mean: pre: 2.41; SD: 1.07 versus post: 3.37; SD: 0.94). In the latter we saw the highest difference between the pre and post survey (difference pre vs. post: 0.96), whereas regarding “student centered learning,” the smallest increase was mentioned after the course (difference pre vs. post: 0.3.32) (Figure 4).

#### 3.2.1 Global items within the core competencies

**Figure 5:**
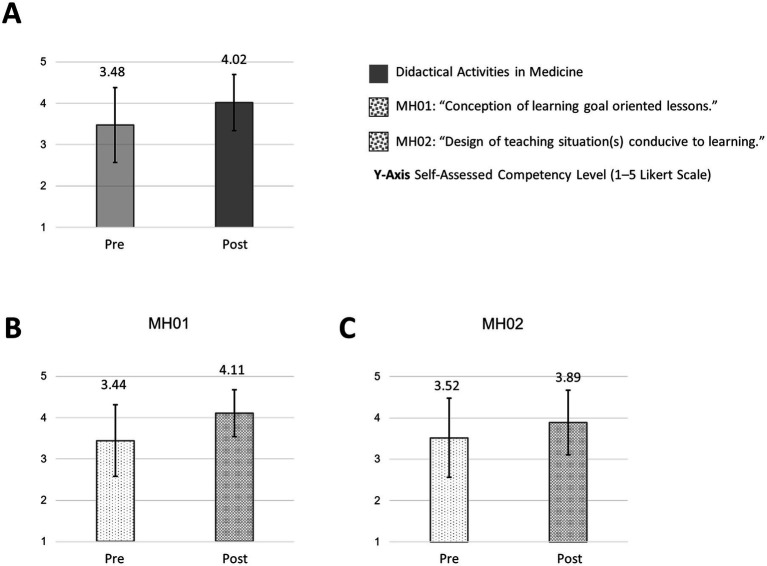
Didactical activities in medicine and subareas pre/post “How to teach pediatrics.” The results show the self-perceived teaching competencies in “didactical activities in medicine” **(A)** and subareas **(B,C)** pre/post our teaching course using the FKM_L questionnaire. The colored bars (pre = transparent/light colored/dotted, post = strong colored/dotted) show the mean values based on a five-point Likert scale with 1 indicating low level of approval, meaning low competency level and 5 indicating a high level of approval, therefore high level of competency. Narrow lines represent the standard deviation. *n* = 9.

The FKM_L questionnaire divides the core competencies into further global items to query subareas of the competency fields. Thus, in both sub-competencies of “didactical activities in medicine,” we saw a slight increase after the course, both in the “conception of learning goal oriented lessons” (MH01: mean: pre: 3.44; SD: 0.86 versus post: 4.11; SD: 0.57) and in “design of teaching situation(s) conducive to learning” (MH02: mean: pre: 3.52; SD: 0.96 versus post: 3.89; SD: 0.79) (Figure 5).

**Figure 6:**
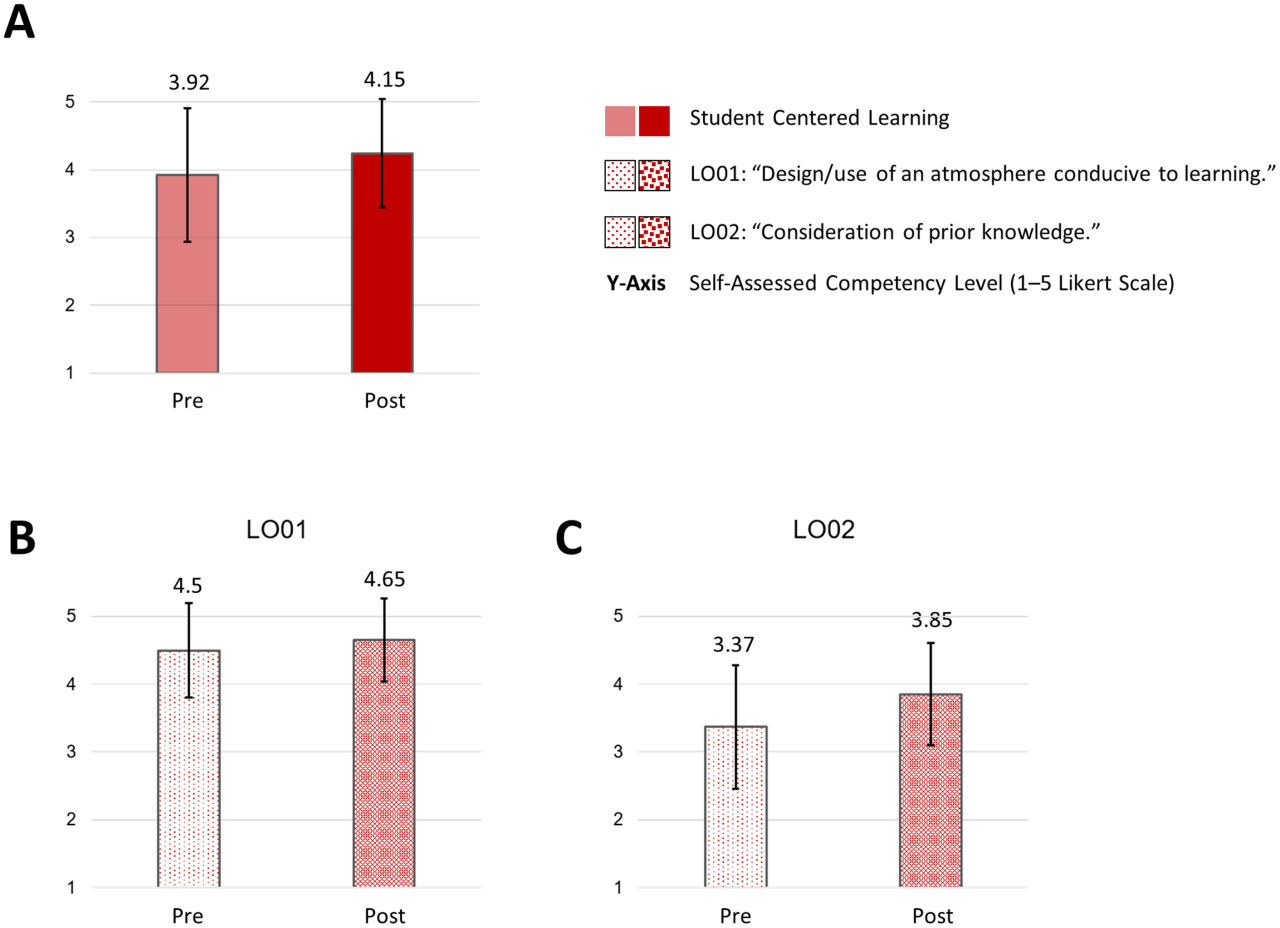
Student centered learning and subareas pre/post “How to teach pediatrics.” The results show the self-perceived teaching competencies in “student centered learning” **(A)** and subareas **(B,C)** pre/post our teaching course using the FKM_L questionnaire. The colored bars (pre = transparent/light colored/dotted, post = strong colored/dotted) show the mean values based on a five-point Likert scale with 1 indicating low level of approval, meaning low competency level and 5 indicating a high level of approval, therefore high level of competency. Narrow lines represent the standard deviation. *n* = 9.

Regarding the subareas of “student centered learning,” there was almost no trend regarding the “design/use of an atmosphere conducive to learning” (LO01: mean: pre: 4.50; SD: 0.69 versus post: 4.65; SD: 0.62). However, a trend towards a slight increase could be observed in “consideration of prior knowledge” (LO02: mean: pre: 3.3.37; SD: 0.91 versus post: 3.85; SD: 0.76) (Figure 6).

**Figure 7:**
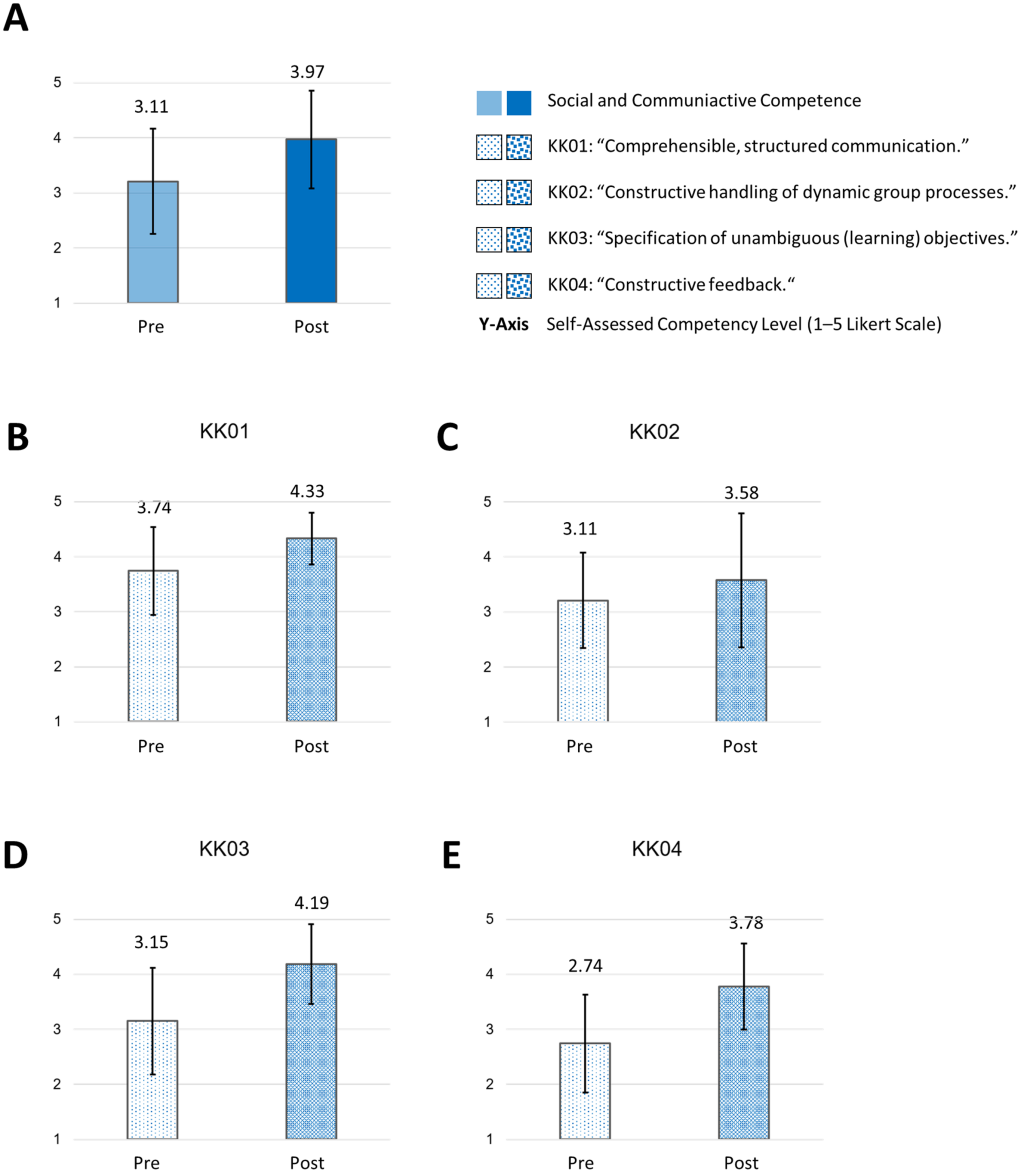
Social and communicative competence and subareas pre/post “How to teach pediatrics.” The results show the self-perceived teaching competencies in “social and communicative competence” **(A)** and subareas **(B–E)** pre/post our teaching course using the FKM_L questionnaire. The colored bars (pre = transparent/light colored/dotted, post = strong colored/dotted) dotted show the mean values based on a five-point Likert scale with 1 indicating low level of approval, meaning low competency level and 5 indicating a high level of approval, therefore high level of competency. Narrow lines represent the standard deviation. *n* = 9.

In the core competency of “social and communicative competence,” participants rated their competency higher in “comprehensible, structured communication” (KK01: mean: pre: 3.74; SD: 0.80 versus post: 4.33; SD: 0.47) and stated a slight improvement of “constructive handling of dynamic group processes” (KK02: mean: pre: 3.11; SD: 0.87 versus post: 3.58; SD: 1.11). Participants reported an improvement in their self-assessed competency in “specification of unambiguous (learning) objectives” (KK03: mean: pre: 3.15; SD: 0.97 versus post: 4.19; SD: 0.72). Additionally, participants rated their competence in giving “constructive feedback” higher after the course (KK04: mean: pre: 2.74; SD: 0.89 versus post: 3.78; SD: 0.79) (Figure 7).

**Figure 8:**
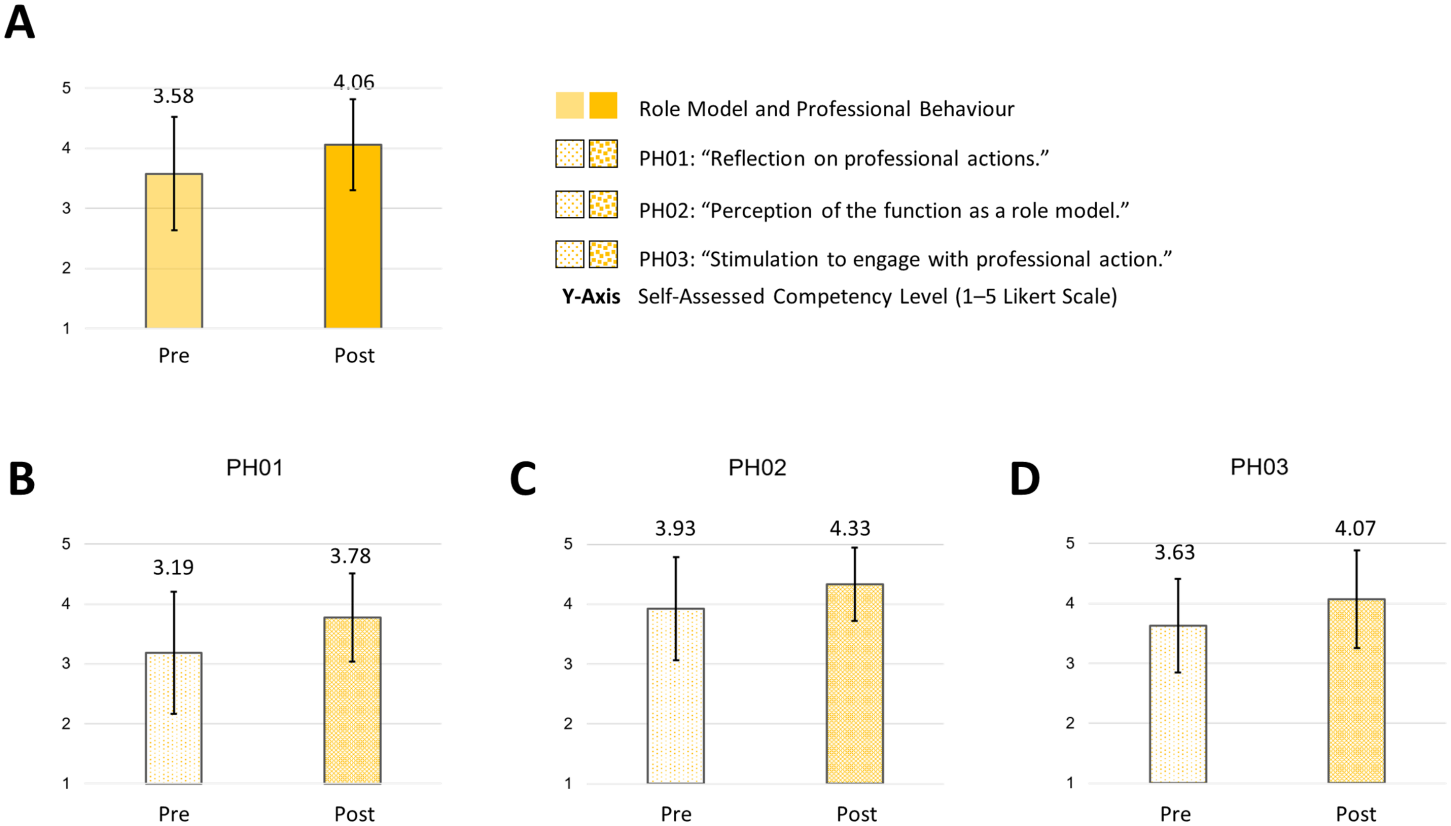
Role model and professional behavior and subareas pre/post “How to teach pediatrics.” The results show the self-perceived competencies in “role model and professional behavior” **(A)** and subareas **(B–D)** pre/post our teaching course using the FKM_L questionnaire. The colored bars (pre = transparent/light colored/dotted, post = strong colored/dotted) dotted show the mean values based on a five-point Likert scale with 1 indicating low level of approval, meaning low competency level and 5 indicating a high level of approval, therefore high level of competency. Narrow lines represent the standard deviation. *n* = 9.

In the competency field of “role model and professional behaviour,” participants reported an increase in the subareas of “reflection on professional actions” (PH01: mean: pre: 3.19; SD: 1.02 versus post: 3.78; SD: 0.74) and “perception of the function as a role model” (PH02: mean: pre: 3.93; SD: 0.86 versus post: 4.33; SD: 0.61). There was a slight increase in “stimulation to engage with professional action” (PH03: mean: pre: 3.63; SD: 0.78 versus post: 4.07; SD: 0.81) (Figure 8).

**Figure 9:**
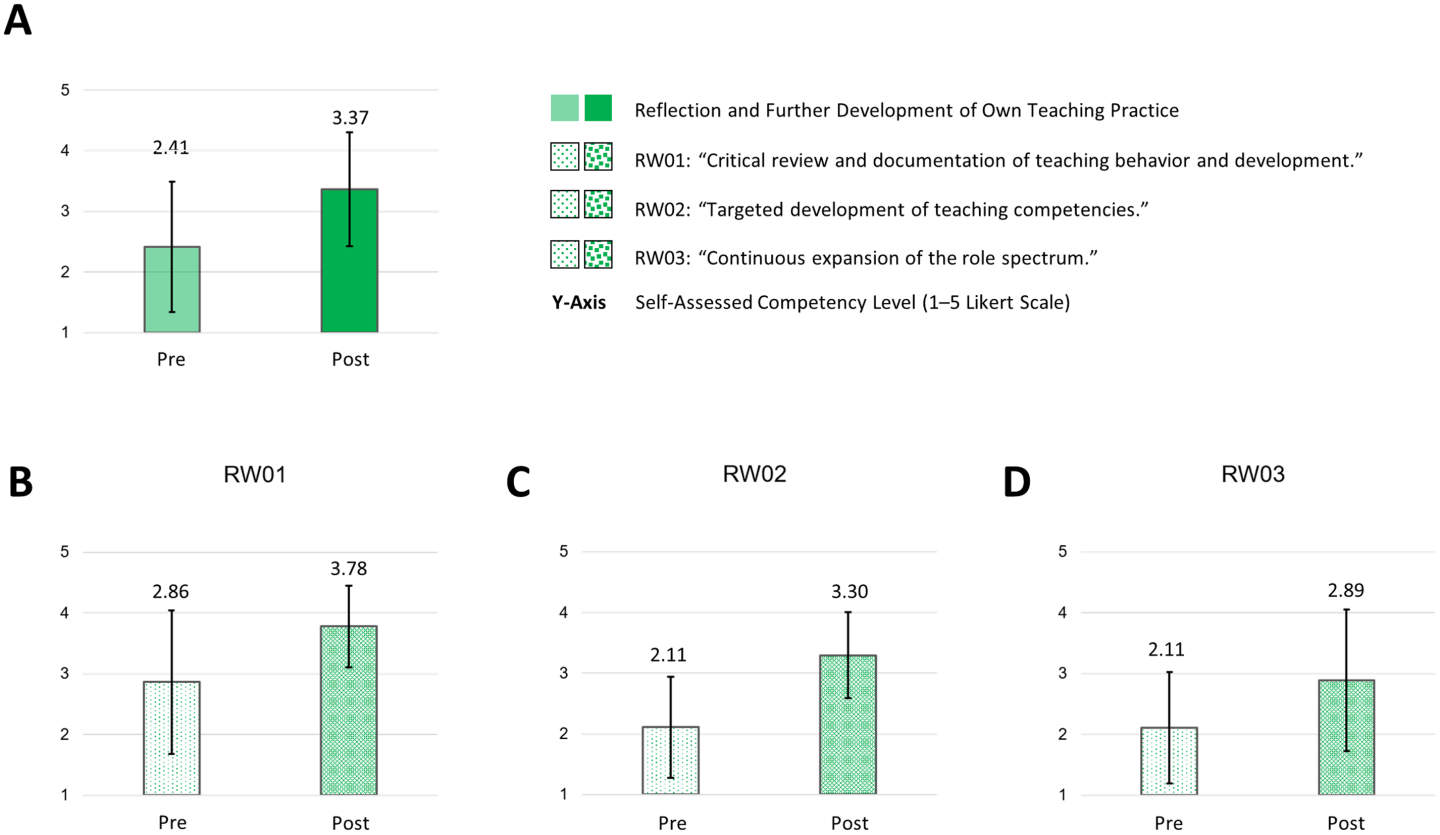
Reflection and further development of own teaching practice and subareas pre/post “How to teach pediatrics.” The results show the self-perceived teaching competencies in “reflection and further development of own teaching practice” **(A)** and subareas **(B–D)** pre/post our teaching course using the FKM_L questionnaire. The colored bars (pre = transparent/light colored/dotted, post = strong colored/dotted) dotted show the mean values based on a five-point Likert scale with 1 indicating low level of approval, meaning low competency level and 5 indicating a high level of approval, therefore high level of competency. Narrow lines represent the standard deviation. n = 9.

The course led to a higher self-assessed competence among participants regarding “critical review and documentation of teaching behavior and development” (RW01: mean: pre: 2.86; SD: 1.18 versus post: 3.78; SD: 0.67) and the “targeted development of [their] teaching competencies” (RW02: mean: pre: 2.11; SD: 0.83 versus post: 3.30; SD: 0.71). Additionally, participants reported to expand their role spectrum after the course (RW03: mean: pre: 2.11; SD: 0.92 versus post: 2.89; SD: 0.78) (Figure 9).

### 3.3 Course evaluation and feedback

**Figure 10:**
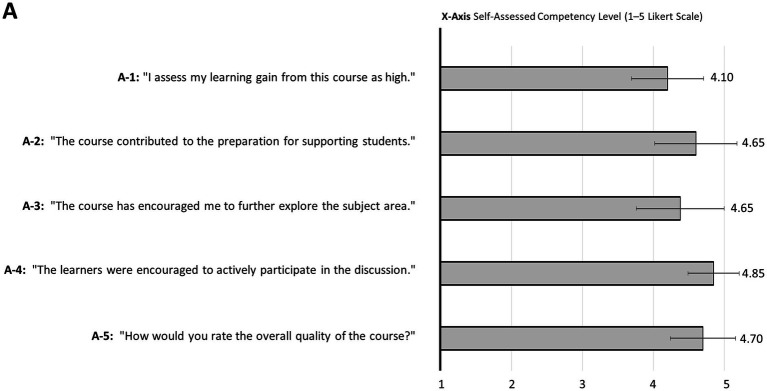
Evaluation. The evaluation of “How to teach pediatrics” by participants is presented. The grey bars show the mean on a five-point Likert scale where 1 = do not agree and 5 = completely agree (A-1 to A-4), and 1 = very poor and 5 = very good (A-5). The standard deviation is represented by narrow lines. n = 20 (A-1, 2, 4, and 5), n = 13 (A-3).

Participants evaluated the course after the second on-site workshop (return rate: n = 20; 76.9%). They stated a high learning gain after participating in the course (Figure 10A-1, Mean: 4.10, standard deviation: 0.51) and estimated the course to be a good preparation for teaching students (Figure 10A-2, Mean: 4.65, standard deviation: 0.57). Furthermore the participants stated that the course encouraged them to further extend their knowledge in learning facilitation for medical students (Figure 10A-3, Mean: 4.65, standard deviation: 0.62). The participants actively took part in the course and the discussion (Figure 10A-4, Mean: 4.85, standard deviation: 0.3.36) and rated the course overall as “very good” (Figure 10A-5, Mean: 4.70, standard deviation: 0.46).

Asked about positive aspects of the course in the free-text comments and in a feedback round at the end of the on-site workshops, participants repeatedly mentioned the “open discussion” and found the “exchange between participants extremely valuable.” They appreciated the “open, trusting atmosphere” and felt that the course was conducted in a “friendly, positive and structured way.” One participant appreciated the “safe learning environment in the course.” Additionally, “helpful ideas for the implementation of teaching techniques and feedback” were positively mentioned, as well as the “opportunity for practical exercises in the course” and introduction to “microteaching techniques” and “teaching with limited time resources.” Moreover, several participants mentioned the areas of “culture of error” and “feedback” as “helpful.”

Asked which aspects of the course could be improved or even be removed from the course the participants found the “e-Learning too extensive.” Moreover, some participants expressed the desire for even “more time dedicated to practical exercises.”

## 4 Discussion

In this study we report on our findings from a faculty development program in postgraduate pediatric training which was attended by 26 participants. The program was designed to bridge the gap in training the learning facilitators for medical students between IPE and non-IPE learning activities in our tertiary pediatric hospital. Based on the framework of core competencies for medical teachers [KLM; (18)], we developed a short course specifically for pediatric residents, which was later expanded to fellows and primary care pediatricians. We measured self-reported teaching competencies, using the validated FKM_L questionnaire (29). We found an increase in self-reported competencies in all areas measured, with some differences that warrant discussion.

Concerning our methodology, we decided to use the FKM_L questionnaire since it was developed based on the same competency framework that we used to define the intended learning outcomes for our course. We hypothesized that this would enable us to assess the effects of our teaching and learning activities in line with the principles of cognitive alignment (26). Secondly, the original questionnaire was developed and validated in German and within the German medical education system. Our study was conducted in Germany, with all participants being German native speakers. Therefore, we omitted possible hindrances that may be caused by non-validated translations, without cross-cultural adaptation (31). However, we made some adaptations to the original version of the FKM_L, removing one core competency and two subareas. These were related to systems-based learning/teaching and taking student exams. The reason for these adaptions was that the target audience for our course were medical doctors involved in day-to-day clinical teaching at the bedside, both individually and in small groups. To increase meaningfulness and reduce cognitive load for participants, we decided to focus on core competencies that are necessary for this particular area of learning facilitation (32). The second focus was on aligning interprofessional and non-interprofessional hands-on learning facilitation, rather than revising curricula or student exams. We therefore selected the five core competencies of the KLM framework the overlap with proposed frameworks for learning facilitators in IPE and adapted the questionnaire accordingly. We decided to apply a retrospective pretest (RPT) methodology for collecting data. This means we made participants complete the questionnaire retrospectively at the end of the course, for both the pre and post assessment. This decision was based on exisiting evidence, that traditional pre/post assessments in interventions like ours are prone to response-shift-bias (33). Moreover, data from the Stanford Faculty Development Program indicate that for training clinical teachers, RPT showed better correlation with housestaff and student evaluation and traditional pre/post comparisons (34, 35). Since the context of our program was similar to the Stanford program, we argue that our choice is supported by existing evidence in the literature.

As for course evaluations results, we observed a high level of motivation and participants positively mentioned open discussions. These factors likely contributed to a positive learning environment. A positive learning environment refers to Level 1 “Reaction” in Kirkpatrick’s Four-Level Model and states the importance of enjoyment of a learning activity (36). Participants were directed towards their own prior experiences as learners, which helped them relate to the course content.

Concerning the results we saw an increase in self-reported competencies in all domains, referring to the Level 2 “Learning” in Kirkpatrick’s model. Generally this does not come as a surprise, since self-reported competencies after a teaching and learning activity tend to increase (37). Still, these findings underline that we were able to address the intended learning outcomes on a global level and point towards a positive influence concerning core medical teaching competencies in our tertiary pediatric hospital.

Taking a closer look at ways how our course influences self-perceived core medical teaching competencies in pediatric residents, we found the biggest increase in self-perceived competency in “Reflection and further development of own teaching practice.” This increase comprised items as “development of own teaching competencies” and “critical reflection of own teaching.” The specific role of a teachers is summarized in the “Scholar” domain of the widely accepted CanMEDs framework (38). However, reflecting on this particular role and its continuous development has not been widely implemented into postgraduate medical training. Therefore our findings are in line with results from other faculty development programs for junior health professionals (20).

We found the smallest difference in pre/post-assessment for the area of “Student centered learning.” One possible explanation is the fact that participants rated this competency higher than in all other core competencies in pre-assessment. Participants were rather young and learner-centered approaches have become more widespread in the last 20 years, so participants are likely to have experienced some learner-centered education themselves (39). Considering the literature, Tipton et al. faced a comparable effect after their residents-as-teachers course with no significant effect in the “ability to create a positive learning environment” with participants starting from a high level (3.73 on a 5-point Likert scale) (40).

In addition to the broader trends, it is important to consider the implications of the changes observed in specific competency areas. For example, competencies related to medical didactics, such as defining learning objectives and ensuring constructive alignment, showed substantial improvement. These concepts, though fundamental to teaching, are often new to residents transitioning from learners to educators. This emphasizes the need for faculty development programs to introduce and reinforce these essential teaching principles.

The increased competency in social and communication skills, particularly in feedback techniques, also merits attention. Practicing feedback techniques were one of the aspects of the course influencing self-perceived competencies in particular. Effective feedback is a critical component of medical education, and the course’s focus on practical feedback strategies likely contributed to the observed gains. These findings are consistent with other studies highlighting the importance of feedback training in improving teaching outcomes (37). The trends of our course we see in the post-course measurement is comparable to other residents-as-teachers programs regarding “giving feedback,” “I am skilled giving feedback” or “providing effective feedback” (40–43).

Despite the immediate positive outcomes in level 1 “reaction” and 2 “learning,” the sustainability and impact (Kirkpatrick levels 3 and 4) of these effects remains uncertain. Participants cited time constraints as a barrier to implementing teaching practices learned in the course. This challenge is well-documented in the literature, with time pressures often limiting the ability of healthcare professionals to engage fully in teaching activities in a long-term view and learned teaching techniques get lost over time (40, 41). While our course provided a solid foundation, its long-term impact on teaching practices will depend on ongoing support and reinforcement.

Strengths of this study include a clear theoretical framework that the course was based on, with a validated questionnaire available for measuring outcomes (18, 29). The theoretical framework was used to inform constructive alignment to ensure that intended learning outcomes, teaching and learning activities and assessment tasks were in line (26). The study addresses a highly relevant topic, adding some new aspects to the existing body of evidence for residents as teachers and how to improve their readiness for teaching.

There are several limitations to this study which need to be taken into careful consideration: The FKM_L questionnaire relies on self-reported outcomes only, which may constitute a significant bias (44). It would be desirable to achieve a more objective way of measuring residents’ teaching competencies. Furthermore, as a result of consistent process evaluation and adaptation, the nature of the course and its participants changed slightly over the time of data collection. This was a natural effect of ongoing quality improvement efforts and changes in staff but should be taken into account when interpreting the data. Sample size is small (*n* = 9) and does not allow for any meaningful statistical analysis, other than of descriptive nature. One reason for this was given by participants as they felt they did not have sufficient time to complete both course evaluation and FKM_L during the course itself. Qualitative data are limited to short answers to open-ended questions and do not allow for extensive exploration of motives for learning success (or the lack thereof). Data interpretation is finally limited by the single-center nature of this study. Some of the effects observed might be due to local circumstances and not be generalizable to other contexts.

To overcome some of these limitations and improve quality of data, some measures have been put into place and will yield in new results in the future: To allow for more time for answering questionnaires during the course, adaptations have been made to the course program. Due to changes in staff policy, it will be possible to run the course twice a year with up to 15 participants each, allowing for more data to be collected in a shorter time. Most importantly, participants will re-assess their teaching competency again when teaching a group of 8 medical students in the two-week 5^th^ pediatrics course. At the same time, data will be collected from student evaluation which is also based on a validated questionnaire. This will allow for a comparison of self-reported teaching competency and assessment by students and might lead to a more objective way of measuring teaching competency and progress.

In conclusion, our study demonstrates that a well-structured faculty development program, grounded in a theoretical framework and aligned with core teaching competencies, can provide an opportunity to enhance the teaching abilities of pediatric residents at a tertiary pediatric center. While the immediate effects are promising, ongoing efforts are needed to ensure the sustainability of these improvements and to explore more objective measures of teaching competency in our context. The small sample size allows only for a descriptive analysis and limits generalizability of our data. Nonetheless those kind of faculty development programs for learning facilitators might allow for students to experience a well-founded level of learning facilitation outside of IPE teaching and learning activities. By continuing to refine and expand our program, we hope to contribute to more effective and impactful faculty development in mono- and interprofessional education.

## Data Availability

The raw data supporting the conclusions of this article will be made available by the authors, without undue reservation.
