# Supplementary material for: Residents as learning facilitators inside and outside of interprofessional education: a faculty development program in postgraduate pediatric training
Source: Front Med (Lausanne). 2025 Feb 6;12:1491177. doi: 10.3389/fmed.2025.1491177 (PMC11839824; doi:10.3389/fmed.2025.1491177)
Supplement: Supplementary file 1 [file Table_1.DOCX]

**Supplementary Material**

## Participants

|  | Number of participants (percent) |
| --- | --- |
| Participants first course (2021)  *Residents*: | 8 (100,0%)  8 (100,0%) |
| Participants second course (2022)  *Residents*: | 7 (100,0%)  7 (100,0%) |
| Participants third course (2024):  *Residents:*  *Fellow:*  *Primary Care Pediatricians:* | 15 (100%)  6 (40,0%)  1 (6,7%)  4 (26,7%) |
| Total participants | 26 (100,0%) |
| Available for the Course Evaluation | 26 (100,0%) |
| Return rate: Course Evaluation | 20 (76,9%) |
| Available for the pre/post self-assessment via FKM_L | 22 (84,6%) |
| Return rate: Pre/post self-assessment via FKM_L | 9 (40,9%) |
| Age: Pre/post self-assessment via FKM_L  25-30 years:  31-35 years:  36-40 years:  Older than 41 years: | 7 (77,8%)  1 (11,1%)  0 (0,0%)  1 (11,1%) |
| Gender: Pre/post self-assessment via FKM_L female | 4 (44,4%) |

Supplementary Table 1: The table shows the composition of the participants and, therefore, the cohort of t
